# Obesity and Selected Allergic and Immunological Diseases—Etiopathogenesis, Course and Management

**DOI:** 10.3390/nu15173813

**Published:** 2023-08-31

**Authors:** Bartłomiej Morąg, Patrycja Kozubek, Krzysztof Gomułka

**Affiliations:** 1 Faculty of Dentistry, Wroclaw Medical University, 50-425 Wrocław, Poland; bartlomiej.morag@student.umw.edu.pl; 2 Student Scientific Group of Adult Allergology, Wroclaw Medical University, 50-369 Wrocław, Poland; 3 Clinical Department of Internal Medicine, Pneumology and Allergology, Wroclaw Medical University, 50-369 Wrocław, Poland; krzysztof.gomulka@umed.wroc.pl

**Keywords:** obesity, asthma, atopic dermatitis, psoriasis, allergic rhinitis, food allergy, complications

## Abstract

Obesity is a global problem. It affects every age group and is associated with many negative health effects. As an example, there is a relationship between obesity and allergic and immunological diseases, such as asthma, psoriasis, food allergies, allergic rhinitis and atopic dermatitis. Obesity undeniably affects their development. In addition, it causes adverse changes in the course and response to therapy in relation to patients without excessive body weight. The treatment of diseases associated with obesity is difficult; drugs are less effective and must be used in higher doses, and their use in patients with obesity is often associated with higher risks. The main form of treatment of all obesity-related diseases is a change in eating habits and increased physical activity, which leads to a decrease in body fat mass. The positive effect of reducing BMI has been confirmed in many independent studies. This paper reviews various types of research documents published since 2019. It aims to systematize the latest knowledge and highlight the need for further research for effective and sustainable treatment options for obesity, its complications and obesity-related diseases.

## 1. Introduction

Obesity affects more than 2 billion people worldwide, and if the upward trend continues, 38% of the world’s adult population will be overweight and 20% will have obesity by 2030. The type of diet has a very significant impact on the calories supplied. Highly processed foods are known to contain large amounts of calories of little nutritional value and contribute to increasing rates of obesity. These days, in the Americas, the so-called “Western diet” became popular. It consists of highly processed foods and fast foods. This diet provides a large amount of calories with little nutritional value. High amounts of saturated fatty acids (high-fat diet—HFD) have a pro-inflammatory effect on dendritic cells and macrophages [1]. Obesity is diagnosed with a body mass index (BMI) above 30 and predisposes to many diseases: metabolic, cardiovascular, psychological, neurological, osteoarticular and hepatobiliary. It is also connected to the development of cancer and diseases of an allergic and immunological origin [1,2]. Adipose tissue is one of the main secretory organs. It is estimated that it is responsible for the release of over 600 different adipokines, including the immunomodulating leptin, resistin and ghrelin [3,4]. Adipokines are involved in inflammatory and autoimmune diseases by influencing innate and acquired immune responses. This paper presents the influence of obesity on the development, course and management of the following diseases: asthma, atopic dermatitis, psoriasis, allergic rhinitis and food allergy [5].

The main aim of the work is to highlight the relationship between obesity and allergic and immunological diseases, and their pathogenesis, development and course. It is necessary to further expand knowledge on this subject and create ways to improve the quality of life of patients and forms of effective treatment in the case of obesity associated with other diseases.

The article is an attempt to collect and systematize the latest knowledge. It was written using English-language publications available in the PubMed database with the publication date from 2019 to July 2023. Searched topics are as follows: obesity-related asthma (697 results), atopic dermatitis and obesity (131 results), allergic rhinitis and obesity (121 results), food allergy and obesity (182 results) and psoriasis and obesity (588 results).

## 2. Asthma

Asthma is a heterogeneous, chronic inflammatory disease of the airways with multiple phenotypes [6,7]. Obesity-related asthma is often described on the basis of paediatric patients, because it particularly affects this group of patients [8,9]. Obesity and related hypovitaminosis D (HVD), obstructive sleep apnoea (OSA) and gastro-oesophageal reflux (GER) contribute to its development [10,11,12]. As a result, patients with obesity have an increased risk of developing asthma. In addition, the severity of asthma in this group of patients (especially patients with abdominal obesity) is higher, and exacerbations are more frequent. Moreover, treatment is more difficult and less effective [10,13,14].

### 2.1. Etiopathogenesis

The development of asthma may result from obesity through many mechanisms, both allergic and non-allergic [11,15,16]. One example is persistent low-level inflammation. Obesity is associated with increased levels of adipokines and proinflammatory cytokines, including leptin, tumour necrosis factor alpha (TNF-α) and multiple interleukins (ILs). There is also an environment with reduced oxygen content (mainly as a result of the rapid growth of adipose tissue). Hypoxic adipocytes release monocyte chemotactic protein (MCP-1). This leads to the migration of monocytes to adipose tissue, where they differentiate into M1 macrophages. M1 macrophages contribute to the development of local and systemic inflammatory reactions [17]. Based on the type of predominant immune response, asthma can be divided into two phenotypes. Their comparison is presented in Table 1. Obesity-related asthma is classified as the T2-low type [17,18,19].

Additionally, oxidative stress is induced through several mechanisms. One of them is lipid metabolism. A high concentration of LDL and its metabolites stimulate the secretion of histamine, while a high level of fats in the diet activates the nucleotide oligomerization domain-like receptor protein 3 (NLRP3) inflammasome and increases sputum IL-1b [20].

Diets high in sugar and saturated fat that are low in fibre can lead to obesity. It has been shown that such a diet leads to decreased lung function and increases the symptoms of respiratory diseases [21].

There is also a hypothesis that airway hyper-reactivity in children with obesity may not be caused by excessive body weight but by hormonal changes. Examples are insulin resistance and impaired glucose tolerance [22].

Common genetic changes have also been shown between obesity-related traits and specific asthma subtypes. A significant genetic correlation between BMI and late-onset asthma (age of 16 or older) has been reported [23]. Another study found a common causal genomic region connecting BMI and childhood asthma that was mapped to the AMN gene. IL-6 (produced in high amounts by M1 macrophages in adipose tissue and increasing the risk of developing asthma) has also been shown to connect childhood BMI with childhood-onset asthma [24].

Another possible factor leading to the development of asthma is a change in the gut microbiota especially in early life. In patients with obesity, the microbiome is less diverse [22].

**Figure 1:**
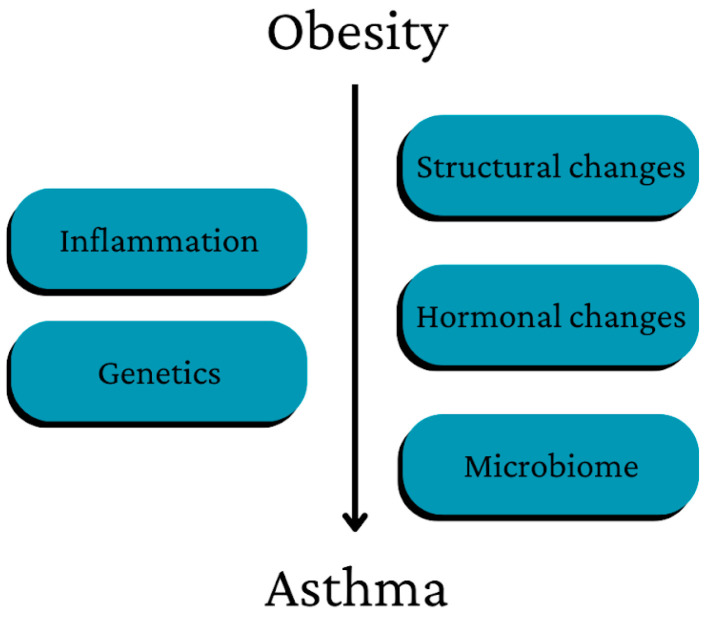
Main pathways of the possible influence of obesity on asthma development [22].

A summary of the main ways in which obesity may contribute to the development of asthma is presented in Figure 1.

The development of obesity itself is also partly caused by asthma [11,25,26]. Examples include the use of systemic corticosteroids and significantly hindered performance of physical activity by patients with asthma, which results in lower calorie burning [11,22].

### 2.2. Course

In patients with obesity, the course of asthma is more advanced than in patients with normal weight. Medications are taken in increased amounts, airway obstruction is increased and exacerbations are more frequent. Attacks occur with greater frequency and are more burdensome for the patient [12,18].

An excessive amount of adipose tissue located around the chest and abdomen (an abnormal waist to hip ratio (WHR)) reduces the movements of the chest and diaphragm, which results in increased respiratory effort and reduced respiratory compliance, both in asthmatic and non-asthmatic patients [25,27]. In children with a too high BMI, there is also a condition called dysanapsis, which consists of an uneven growth of the lung parenchyma and the diameter of the airways. It is associated with a more severe course of asthma [19,22,25]. A spirometry test in subjects with obesity showed a lower forced expiratory volume during the first second of expiration (FEV 1). This result was more noticeable in the group of adult patients compared to children. Forced vital capacity (FVC) and maximum mid-expiratory flow rates (FEF 25–75%) were also lower in overweight patients. Fractional exhaled nitric oxide (FeNO) level assessment may be helpful in determining allergic and non-allergic causes of lower airway hyper-responsiveness [25]. Changes in lung volume and bronchial hyper-responsiveness should also be considered in the diagnosis of asthma. The methacholine challenge test is beneficial [18].

### 2.3. Management

The therapy of obesity-related asthma is difficult—it is associated with an increased supply of medications and more frequent hospitalizations [28]. Treatment should be based on reducing and stabilizing body weight. Studies have shown that a 5–10% decrease in body weight in asthmatic patients significantly improves asthma-related quality of life and asthma control [18]. The Mediterranean diet (high in fish, omega-3 fatty acids, fresh fruits and vegetables) has a protective effect against asthma due to its beneficial effect on inflammation, oxidation and microbial composition [22]. Bariatric surgery has also been shown to be effective [7].

Daily preventative inhaled corticosteroids (ICS) and combined corticosteroids with LABA are less effective in patients with obesity than in patients with a normal BMI. Late-onset obesity-associated asthma is seldom associated with high eosinophilia; therefore, new biologics (such as anti-IgE: omalizumab, and anti-eosinophils: mepolizumab, reslizumab and benralizumab) do not show significant efficacy in the treatment of this group of patients [7,18]. Clinical trials on the use of an anti-interleukin antibody, tocilizumab, in the treatment of obesity-related asthma are ongoing [29]. Vitamin D supplementation is considered an adjuvant treatment [10,12].

### 2.4. Summary

Obesity is a significant factor increasing the risk of developing asthma and a more severe course of the disease. Obesity-related hypovitaminosis D, obstructive sleep apnoea and gastro-oesophageal reflux play a significant role in the development of asthma. In the diagnosis of asthma in patients with excessive body weight, great emphasis should be placed on changes in lung volume and bronchial hyper-reactivity. Fat accumulated in the abdominal and thoracic regions has a negative impact on breathing even in the absence of asthma. Patients with obesity have more frequent exacerbations, severe symptoms and difficult and less effective treatment. The basis of obesity-related asthma treatment should be the reduction in body fat mass. Pharmacological treatment is less effective due to a different type of immune response. As a result, the effect of classic drugs (LABA and corticosteroids) and new biological drugs (e.g., omalizumab, mepolizumab, reslizumab and benralizumab) is not sufficient.

## 3. Atopic Dermatitis

One of the main chronic and recurrent inflammatory skin diseases—atopic dermatitis (AD)—is closely related to obesity [3]. AD is a global problem affecting mainly children and adolescents, although it also affects the older population [30,31]. AD at a young age predisposes to the development of other allergic diseases later in life: allergic rhinitis, asthma and food allergy. This phenomenon is called the atopic march [30]. Atopic dermatitis is manifested by an erythematous rash, dry and cracked skin and intense itching. It significantly affects the quality of life of patients, and predisposes to anxiety, sleep disturbances and depression [30,32].

### 3.1. Etiopathogenesis

Obesity leads to the inflammation of the skin with many forms of manifestation. This is due to the complex interactions between adipocytokines, hormones, fatty acids and mechanical factors [3]. In addition, obesity reduces the barrier function of the epidermis through increased blood pressure and increased sweating. There are also hypotheses about the relationship between obesity and atopic dermatitis caused by genetic factors or the microbiome [32]. The intestinal microbiome is less diverse in people with obesity. The skin microbiome also changes with BMI. An example is Corynebacterium colonization associated with a higher BMI and increased adipogenesis [30]. An increased risk of developing AD has also been demonstrated in children of mothers who had a too high BMI (overweight or obese) before pregnancy [4].

### 3.2. Course

In the course of atopic dermatitis, reduced lipid content in the skin, increased trans- epidermal water loss and a higher pH are observed. Obesity is associated with more severe AD [30]. Resistance to anti-inflammatory treatment is also increased [33]. Additionally, the influence of atopic dermatitis on the development of obesity has been demonstrated [32].

### 3.3. Management

It has been proven that weight loss alleviates symptoms and improves AD treatment outcomes [4,33]. In pharmacological treatment, great hopes are pinned on Peroxisome-proliferator-activated receptor gamma (PPARγ) as a potential therapeutic target. PPARγ is expressed, for example, in adipose tissue and naturally has anti-inflammatory properties (as a result of, inter alia, the suppression of Th17 cells). The thiazolidinedione (TZD) rosiglitazone has been shown to alleviate atopic dermatitis in combination with a high-fat diet (fat lowers PPARγ levels in Th2 cells, leading to inflammation). In addition, a better effect of this medication in combination with dexamethasone than in the case of monotherapy has been described [30,34].

### 3.4. Summary

Atopic dermatitis affecting mainly the youngest patients significantly reduces quality of life. Obesity, both in the mother before pregnancy and in the patient, plays a role in its development. Obesity affects AD through multiple mechanisms: the reduction in the epidermal barrier function, interactions with substances secreted by adipose tissue, genetic factors and changes in the intestinal and skin microbiota. Obesity also has a negative impact on the course of the disease and its treatment. Body weight reduction is suggested as a treatment. Drugs using PPARγ transcription factors as a therapeutic target are also being studied.

## 4. Psoriasis

Psoriasis (PSO) is a chronic, recurrent, papulo-squamous inflammatory skin disease [3]. It can develop at any age—on average, at 33 years of age, and earlier in women. PSO is associated with high physical and mental strain and a shorter lifespan. The most common phenotype is psoriasis vulgaris [35]. PSO classically takes the form of an erythematous plaque covered with silvery scales, the removal of which reveals the Auspitz sign (minor bleeding spots). The size of the plaque can vary. Its edges are the most active area and the lesion expands outwards. The lesions typically occur symmetrically, in the extensor areas of the knees and elbows, on the scalp and in the lumbosacral region [35].

### 4.1. Etiopathogenesis

In the pathogenesis of psoriasis, the following are involved: genetic factors (for example, related to the human leukocyte antigen—HLA), microbial dysbiosis and inflammation and environmental factors such as stress, smoking, alcohol consumption and obesity. A key role is attributed to interleukin 17 and 23 [1,30,35,36]. Some pro-inflammatory mechanisms in psoriasis are consistent with pro-inflammatory mechanisms in obesity. Examples include interferon-γ; interleukin 2, 6 and 12; and tumour necrosis factor (TNF-α) [4,30]. Adipokines (leptin and adiponectin), free fatty acids and adipocytokines (TNF-α, IL-1β and IL-6) are also associated with the pathogenesis of psoriasis.

Serum free fatty acid levels, including oleic and palmitic acids, correlate with the severity of inflammation in psoriasis. High levels of these substances may lead to an exacerbation. Leptin contributes to the low-grade inflammatory state characteristic of obesity, which predisposes to the development of autoimmune diseases. The level of adiponectin, which has anti-inflammatory properties, is reduced in both PSO and obesity.

The subclinical, systemic inflammation observed in patients with obesity can be associated with metabolic endotoxemia. It is an immune response of the patient’s organism to LPS (from the wall of Gram-negative bacteria) circulating in the serum. LPS passes from the intestinal lumen into the circulation. It is worth emphasizing that patients with obesity present weakened function of the intestinal epithelial barrier, which is associated with an increased permeability of small molecules through the intestinal wall. Moreover, the level of two marker proteins of changes in the intestinal barrier, zonulin and LPS-binding protein, is elevated in people with too high body weight. The study also found twice the increase in intestinal permeability after loading with saturated fatty acids in relation to people with a normal BMI. This was associated with systemic and enteric inflammation [1].

### 4.2. Course

Obesity negatively affects the course of psoriasis and its treatment [4]. Similarly, a diet high in saturated fats, including oleic and palmitic acids, and their high serum levels, is closely correlated with the exacerbation of psoriatic dermatitis. A significant impact of psoriasis on the development of obesity was also shown—in children, it was greater than in the case of atopic dermatitis [1].

PSO and obesity are also associated with reduced quality of life and negative psychosocial impact. As a result, in the case of the coexistence of both diseases, the mental state of patients is more disturbed and depression occurs with higher frequency [1,37].

### 4.3. Management

Weight loss is a proven factor in reducing the severity of psoriasis and mitigating its course. Some studies also suggest a variety of dietary interventions, including eating foods that inhibit inflammatory pathways, involving polyunsaturated fatty acids, vitamin D and B12, short-chain fatty acids, dietary fibre, probiotics and selenium [1,4,38]. The most common nutrients that contribute to psoriasis are summarized in Table 2.

Pharmacological treatment is more difficult, less effective and burdened with more side effects, mainly cardiometabolic complications (mainly as a result of the usage of higher doses of pharmaceuticals) [1]. Infliximab and ustekinumab are considered the most useful drugs. Patients can also benefit from apremilast, one of the side effects of which is weight loss, desirable in this group of patients [40]. Clinical trials of pharmaceuticals have shown the antipsoriatic effect of thiazolidinedione [30]. Positive results of therapy have also been found in the case of bariatric surgery and treatment with glucagon-like peptide-1 analogues (for example, liraglutide and semaglutide) [1].

### 4.4. Summary

Psoriasis is a disease that reduces the quality of life of the patient in many ways, including physical and mental burden. The pathogenesis of PSO involves many factors, including genetic and environmental determinants. It is associated with obesity, which worsens the course of PSO and makes treatment more difficult. There is also the opposite effect when psoriasis influences the development of obesity. The therapy emphasizes the significant impact of weight loss on the severity of symptoms. A variety of dietary, surgical and pharmacological interventions are available with proven efficacy. In pharmacological treatment, the following may be useful: etanercept, a blocker of tumour necrosis factor alpha, and anti-inflammatory analogues of glucagon-like peptide-1.

## 5. Allergic Rhinitis

Obesity is a factor that increases the risk of atopic allergic diseases in children [41]. One example is allergic rhinitis (AR). Studies have confirmed its relationship with excessive body weight in the paediatric population, but no such relationship was found in adults [42]. Allergic rhinitis is an allergic disease that has been increasing in frequency for years [43]. Although it does not affect mortality, it is a disease that reduces the quality of life of patients through poor sleep quality, fatigue and stress [43,44,45].

### 5.1. Etiopathogenesis

Obesity leads to changes in the immune system. This is mainly due to the increased level of leptin and IL-1β in the serum. Leptin may induce active inflammation in allergic rhinitis by activating IL-1β (an important marker of the activation and exacerbation of allergic diseases) [43]. Other mechanisms may lead to reduced tolerance to antigens and an increased risk of allergy due to the skewness of the immune system towards a Th2 cytokine profile [43,44].

### 5.2. Course

Allergic rhinitis is a disease that affects the nasal mucosa. It is manifested by its congestion, sneezing, nasal discharge and itching. It is also associated with reduced concentration, sleep disorders, snoring and mood deterioration [43,46]. Obesity increases the risk of exacerbation and persistence of AR symptoms. A decrease in BMI in children is associated with a decrease in the concentration of leptin and IL-1β and alleviation of AR symptoms [45].

There are also hypotheses that obesity is a factor that increases the negative impact of air pollution on the course of allergic rhinitis. It includes increasing nasal patency and stronger inflammation of the mucous membrane [45].

AR may also increase the risk of developing obesity due to reduced physical activity and side effects of treatment with selected chemotherapeutics [43].

### 5.3. Management

As in other allergic diseases, weight loss reduces inflammation and has a positive effect on the course of allergic rhinitis. Pharmacological treatment is not significantly different from that used in children with a normal BMI [43].

### 5.4. Summary

Allergic rhinitis is a common disease that negatively affects the mental health and quality of life of the patient. The relationship between obesity and AR has been found in the paediatric population. Obesity, through many mechanisms, increases the inflammatory process in the body and changes the functioning of the immune system. This makes the symptoms of allergies worse and last longer. In therapy, BMI reduction is effective as it leads to a reduction in inflammation and stabilization of the course of AR.

## 6. Food Allergy

Food allergy is an inflammatory disease that is associated with a specific allergen. It is common and has a significant impact on the patient’s life [47]. Food allergies affect both children and adults. A new allergy can become active at any age, mainly at early childhood proceeding obesity [48]. The eight most common food allergens are peanuts, eggs, fish, milk, tree nuts, wheat, crustacean shellfish and soy [49]. In some cases, obesity affects the risk and course of new food allergies as a result of, inter alia, the low-grade inflammatory state and the non-specific activation of the immune system [47].

### 6.1. Etiopathogenesis

Obesity and the associated high-fat diet lead to the inflammation of the intestines. The number of macrophages, T-lymphocytes, intraepithelial lymphocytes and mature dendritic cells is increased. These cells produce pro-inflammatory cytokines (including TNF-α, IFN-γ and IL-1β), which contribute to the development of inflammation in the intestinal lamina propria and epithelial compartments. Ultimately, it comes down to a weakening of the intestinal barrier function [50]. Inflammation is associated with a reduced tolerance of the immune system to allergens. Obesity and HFD change the composition of the intestinal microbiome and lead to an increased permeability of the intestinal wall [47]. The influence of obesity and a high-fat diet on PPARγ receptors is important. There is a decrease in their expression, phosphorylation and epigenetic events, which leads to impaired receptor function. PPARγ has an anti-inflammatory function by inhibiting pro-inflammatory genes. When their expression is reduced, genes are inhibited and the inflammatory effect on the body increases. All the processes described above lead to the development of food allergy [34,47].

### 6.2. Course

Food allergy manifests itself very diversely, from minor symptoms of discomfort or pruritus, through moderately severe symptoms—diarrhoea, vomiting, shortness of breath and urticaria—to life-threatening anaphylaxis [47]. In studies on mice, the relationship between obesity-induced hyperglycaemia, the aggravation of food allergy and the impairment of oral tolerance induction has been proven (oral tolerance is a local and systemic immune unresponsiveness to orally ingested antigens such as food) [50].

### 6.3. Management

The management of food allergy strictly depends on the intensity of the symptoms. The basis of therapy is avoiding products that cause an allergic reaction and emergency treatment in case of symptoms. Weight loss, the treatment of hyperglycaemia and dietary changes (for example, a reduction in foods rich in fatty acids) are beneficial to the patient [47]. It is important to try to prevent the development of food allergy by introducing allergenic foods during the weaning period [51].

### 6.4. Summary

Food allergy is a common immune-mediated inflammatory disease with highly diverse symptoms. Obesity, hyperglycaemia and HFD are associated with the development and course of food allergy. This happens through many mechanisms. The most important of these is the persistent, low-grade inflammation characteristic of obesity and associated with many adverse phenomena. In allergy therapy, the most important thing is to avoid allergens. Weight loss is also useful as it reduces inflammation.

## 7. Discussion and Conclusions

This paper summarizes and systematizes the latest knowledge on diseases of an allergic and immunological origin: asthma, atopic dermatitis, psoriasis, allergic rhinitis an food allergy.

Obesity is a global epidemic that is constantly evolving. It shows indisputable influence on the development, course and management of various diseases. Its pathogenesis consists of many complex mechanisms. The process of building up fat is called adipogenesis. It can take the form of hypertrophy, which is an increase in the size of existing adipocytes, and hyperplasia consisting of the formation of new adipocytes [1,3].

Obesity is characterized by low-grade systemic inflammation. Under normal conditions, adipocytes produce hormones and cytokines called adipokines. Those substances take part in the regulation of the body’s homeostasis system. The hypertrophy of white adipose tissue disturbs the activity of adipocytes. They produce higher amounts of pro-inflammatory adipokines, resulting in higher levels of inflammation, also called metainflammation. Hypoxia and intracellular oxidative stress are also important in its pathogenesis [5]. Constant inflammation leads to the increased risk of developing many diseases, including allergic and immunological ones [1]. Reducing the BMI in the population would lead not only to a reduction in the number of people suffering from obesity but also other systemic diseases.

Asthma is an example, in which pathogenesis is influenced by obesity through changes in the microbiome, disturbed hormonal balance, structural changes in the body and continuous low-grade systemic inflammation [22].

In the case of allergic rhinitis, the relationship between the risk of its occurrence and obesity in the elderly population has not been proven but in children such a relationship exists [42]. It can be concluded that obesity most severely affects younger children and causes the greatest changes in their organism. Additional therapy in this group would provide the best results.

The main method of therapy should consist of a change in diet and increase in physical activity. These measures should lead to the decrease in body weight and reduced production of pro-inflammatory substances by AT. As a result of which, systemic inflammation would be reduced and quality of life would be improved [5].

There are several limitations to this study. First and foremost, the way of selecting articles and process of data extraction were performed subjectively. Although, the authors made a big effort to choose articles of the best quality and latest knowledge. Another limitation is the omission of non-English articles, which could have limited the search strategy. The last limitation is a highly limited date of publication, which could have led to the oversight of crucial data.

More research is needed and the relationship between obesity and other diseases should be studied for further understanding. In the future, research should focus on the ways to improve the quality of life of these patients and introduce effective treatment that is not an excessive burden for the patient and does not bring major side effects. It is also important to promote obesity prevention and a healthy diet as a form of reducing the risk of developing various systemic diseases.

## Figures and Tables

**Table 1:** Comparison of two phenotypes of asthma based on the type of immune response [17,18,19].

T2-High	T2-Low
Overexpression of the immune type 2 ways (for example IL-4 and IL-13 genes)	Immune type 2 response is not overexpressed (overexpression of genes related with the inflammasome and superfamilies of interferon and tumour necrosis factor)
High sputum eosinophilia	High sputum neutrophilia
High exhaled nitric oxide fraction	-
High efficiency of omalizumab, mepolizumab, reslizumab and benralizumab	Low efficiency of omalizumab, mepolizumab, reslizumab and benralizumab

**Table 2:** The most common nutrients with an effect on psoriasis [1,39].

Nutrient	Main Dietary Sources	Effect on Psoriasis
sucrose	sweetened beverages, sweets, fast food	possible exacerbation of psoriasis
n-3 PUFAs	fish	anti-psoriatic effects
SFAs, such as palmitic acid or stearic acid	butter or red meat	exacerbation of the IMQ-induced psoriasiform dermatitis
n-6 PUFA linoleic acid	vegetable oils or margarines	possible promotion of psoriasis
vitamin D	Dietary intake (tuna, cod liver oil, beef liver, salmon, cheese, sardines, egg) and synthesis in skin	anti-psoriatic effects
selenium	fish, shellfish, eggs, grains	amelioration of psoriasis, anti-oxidative and immunoregulatory properties
vitamin B12	fish, oyster, clam, liver (beef, pork or chicken liver)	amelioration of psoriasis and anti-inflammatory, anti-oxidative effects
SCFAs (e.g., acetate, butyrate, propionate)	dietary fibre (in the process of fermentation)	amelioration of psoriasis, systemic and intestinal anti-inflammatory effects
alcohol (ethanol)	alcoholic drinks, food containing alcohol	promotion, exacerbation of psoriasis

## Data Availability

Data sharing is not applicable as no datasets were generated or analysed during the current study.
